# Reliability of Paper-Based Routine Documentation in Psychiatric Inpatient Care and Recommendations for Further Improvement

**DOI:** 10.3389/fpsyt.2019.00954

**Published:** 2020-01-14

**Authors:** Daniela Fröhlich, Christin Bittersohl, Katrin Schroeder, Daniel Schöttle, Eva Kowalinski, Stefan Borgwardt, Undine E. Lang, Christian G. Huber

**Affiliations:** ^1^ Universitäre Psychiatrische Kliniken Basel, Universität Basel, Basel, Switzerland; ^2^ Klinik und Poliklinik für Psychiatrie und Psychotherapie, Universitätsklinik Hamburg-Eppendorf, Hamburg, Germany

**Keywords:** basic documentation, psychiatric routine documentation, routine data, patient files, data quality

## Abstract

**Background:** Health services research is of increasing importance in current psychiatry. Therefore, large datasets and aggregation of data generated by electronic routine documentation due to legal, financial, or administrative purposes play an important role. However, paper-based routine documentation is still of interest. It remains relevant in less developed health care systems, in emergency settings, and in long-term retrospective and historical studies. Whereas studies examining the reliability of electronic routine documentation support the application of routine data for research purposes, our knowledge regarding reliability of paper-based routine documentation is still very sparse.

**Methods:** Basic documentation (BADO) was completed on paper forms and digitalized manually for all inpatients of the Department of Psychiatry and Psychotherapy, University Hospital Hamburg-Eppendorf, Germany, treated within the time period from 1998 to 2006. Four hundred twelve cases of first-episode psychosis patients were chosen for comparison with clinical data from paper-based patient files. The percentage of missing information, the percentage of correct classifications, sensitivity, and positive predictive value were calculated for all applicable variables.

**Results:** In eight cases (1.9%), a BADO form was available, but was not filled in. In 37 cases (7.0%), the patient files were lost and could not be obtained from the centralized archive. Routine data were available for all other cases in 20 (58.8%) of the examined 34 variables, and the percentage of missing data for the remaining variables ranged between 0.3% and 22.9%, with only the variables education and suicidality during treatment having more than 5% missing data. In general, the overall rate of correct classifications was high, with a median percentage of 86.4% to 99.7% for the examined variables. Sensitivity was above 75% for eight and <75% but above 50% for six of the examined 17 variables. Values for the positive predictive value were above 75% for nine and <75% but above 50% for three variables.

**Conclusion:** In summary, paper-based routine documentation reaches acceptable reliability, but this is dependent on the chosen documentation categories and variables. Based on the present findings, paper-based routine documentation can indeed be used for quality management, organizational development, and health services research. Its limitations, however, have to be kept in mind.

## Introduction

Health services research, the use of routine data, secondary analyses of public as well as proprietary datasets, and the application of “big data” strategies to answer research questions are of increasing importance in current psychiatric research (1–3). The use of routine data can expand scientific knowledge beyond answers given by randomized controlled trials (RCTs) with strict in- and exclusion criteria, patient samples omitting severely ill populations because of their inability or unwillingness to provide informed consent, and study protocols differing from clinical day-to-day practice (2–4). While RCTs are providing us with knowledge of a high evidence level, health services research can add information from cost-effective naturalistic studies with large sample sizes and better generalizability, leading to a better translatability into clinical practice (3).

In addition of being useful for health services research, clinical routine data serves multiple other purposes. It is the basis for cost-effective and timely controlling of clinical processes, quality management, and financial as well as organizational development (5). Furthermore, use of common instruments and variables allows benchmarking across different health care providers. Attempts to implement a common instrument for the collection of routine data in German hospital psychiatry have a long history, from the standard documentation form (“Normalschema”) by Flemming in 1846, continuing to the basic documentation (BADO) of the German Association for Psychiatry and Neurology (DGPN-BADO) by Dilling in 1982, and to the BADO of the German Association for Psychiatry, Psychotherapy, and Neurology (DGPPN-BADO) by Cording in 1995 (6, 7).

These paper-based instruments have often been replaced by digital routine documentation (8–10) or data extraction from structured clinical databases used for electronic patient files (11–15). Large datasets, generated in hospitals due to legal, financial, or administrative purposes, from insurance companies (16) or federal offices (17) enable data collection and aggregation at a much broader scope (3, 4). Nevertheless, paper-based routine documentation and paper-based patient files are still relevant today. Electronic and paper-based documentation each have different advantages and shortcomings (18, 19). In less developed health care systems paper based documentation remains the key instrument and even in developed health care systems (e.g. in outpatient treatment settings) clinical documentation is often only partly digitalized. Also in settings where data has to be available quickly and documentation is performed under time pressure, e.g. in emergency settings (20), paper-based documentation is the preferred instrument. Last but not least long-term retrospective and historical studies still depend on paper-based documentation (4, 21).

An important limitation for application of routine documentation for health services research is the question of its reliability. Unfortunately, knowledge of the reliability of paper based documentation, e.g. the DGPPN-BADO, is limited despite its repeated application for research (4, 22, 23). Whereas one study is available examining the reliability of electronic routine documentation using an adapted version of the DGPPN-BADO and implementing several methods for the increase of data reliability and completeness (24), this research question remains unanswered for paper-based versions of the BADO. Electronic documentation might be of better data quality due to several reasons. It offers automatic checks for completion of required information, checks for the adequate data type and value range, and can be enforced in a timely manner to avoid a memory bias (3, 21).

### Aim of Study

Paper-based routine documentation is still used in current healthcare settings. It constitutes an important data source for health services research, but its reliability is, at present, unclear. Therefore, the aim of the current study is to examine the reliability of paper-based routine documentation of inpatient cases in psychiatry. Based on the available literature, we hypothesized that reliability of paper-based routine documentation might be poorer compared to electronic routine documentation.

## Methods

Data was derived from patient files and BADO of inpatients of the Department of Psychiatry and Psychotherapy, University Hospital Hamburg-Eppendorf (UKE), Germany, treated within the time period from 1998 to 2006. During this time period, the Department of Psychiatry and Psychotherapy was legally obliged to provide psychiatric inpatient treatment for a specific sector of Hamburg, Germany, an urban catchment area with a population of about 300,000 persons. One hundred fifty-five beds on seven specialized wards were available for inpatient treatment. During the investigation period, a total of 21,614 inpatient cases were recorded, equaling to about 2,300 inpatient cases per year.

### Study Population

The current study analyses data originally collected within the scope of quality management efforts to improve treatment in first-episode psychosis patients. Thus, inpatient cases with a first-time hospitalization in the Department of Psychiatry and Psychotherapy because of a psychotic syndrome during the time period from 1998 to 2006 were analyzed. This quality management data was later made available for research projects. For example, index cases for the MD thesis of Türk (25) and for the studies of Huber et al. (26, 27) were identified using this database (25–27).

All data were recorded during routine treatment, collected as part of internal quality management efforts, and anonymized during extraction. Thus, according to legal regulation, no formal approval from the local ethics committee was required. However, the responsible ethics committee was informed about the structured data collection and had no objections (Ethik-Kommission der Ärztekammer Hamburg, Hamburg, Germany, OB-026/06). In addition, for this kind of retrospective, secondary analysis of routine clinical data, informed consent is in general not obtained and not necessary. Our study did not lead to any disadvantages or harm for the participants, and the identification of single individuals is not possible. The current study was conducted in compliance with all local and national regulations.

### Basic Documentation (BADO)

From 1998 to 2004, an adapted version of the BADO (7) as recommended by the German Association of Psychiatry, Psychotherapy, and Psychosomatics (DGPPN) was used for documentation of routine data (7). From 2005 to 2006, a modified version was used omitting the variables nationality, forced medication, suicidality during admission, suicidality during treatment, and suicide attempt during treatment. A new variable “behavior endangering others” was added in this revision. The physician managing the case at discharge was responsible to complete a paper version of the BADO. A secretary checked if a discharge note and a completed BADO form were available in the patient files. Data were not routinely checked for completeness or quality, but in the case of a completely missing BADO form, the responsible physician was contacted and completion of the form was requested.

### Patient Files

During the observation period, the Department of Psychiatry and Psychotherapy exclusively used paper-based patient files encompassing all information available at entry, treatment-related documentation, and discharge notes. Data of cases matching in- and exclusion criteria were collected by two research assistants (CB and HT) using a database built to mirror BADO variables. To ensure that all information available in the patient files was entered completely and correctly, a random sample of 10% of the cases was re-checked by the research assistant not responsible for data entry of the relevant case.

### Data Management

To ensure comparability with the published literature, an approach according to Jaeger et al. (24) was chosen for the current study. Thirty-four variables were selected for analysis: date of birth, gender (three categories), marital status (four categories), nationality, education (seven categories), occupational situation (seven categories), living situation (11 categories), zip code, diagnosis according to ICD-10 (main and co-morbid diagnoses were considered, and one variable for each diagnostic group from F0 to F9 was coded as either ‘present’ or ‘not present’), admission ward, date of admission, discharge ward, date of discharge, treatment duration, sector patient (three categories), type of admission (five categories), type of entry (three categories), type of discharge (five categories), legal care (three categories), forced medication (three categories), behavior endangering others, suicide attempt in the past (three categories), suicidality during admission (three categories), suicidality during treatment (three categories), and suicide attempt during treatment (three categories).

A coding scheme was created to compare information available from patient files and from the BADO according to Jaeger et al. (24). Polytomous items with different categories were recoded into multiple single variables—one for each category. When multiple answers were possible for an item, these were also recoded into multiple new dichotomous variables.

### Software Used

Information from the paper-based BADO was manually entered in an electronic database (Filemaker Inc., Santa Clara, CA, USA) by a clinical secretary after discharge. For analyses, data was exported to Microsoft Excel (Microsoft, Redmond, WA, USA) and imported to PASW Statistics 18.0 (Chicago, IL, USA). Statistical analyses were conducted using PASW Statistics 18.0.

### Statistical Analyses

The following measures were calculated: percentage of missing information in the patient file per variable; percentage of missing information in the BADO form per variable; percentage of correct classifications, i.e. rate of correctly positive and correctly negative coded ratings, calculated by dividing the frequency of correct ratings by the number of total available ratings; sensitivity, i.e. probability that an information present in the patient files is correctly coded in the BADO, by dividing the frequency of correct positive ratings in the BADO by the number of all occurrences of the information in the patient files, and the positive predictive value, i.e. probability that an information coded in the BADO is indeed present in the patient files, by dividing the frequency of correct positive ratings in the BADO by the number of all positive ratings in the BADO. For some variables (date of birth, zip code, having a main or co-morbid diagnosis of one of the ICD-10 diagnosis groups, admission ward, date of admission, discharge ward, date of discharge, and treatment duration) the frequency of correct and false positive and negative ratings cannot be determined, and a simplified assessment had to be used (ratings in the BADO and the patient file agree/disagree). Thus, for these parameters, sensitivity and the positive predictive value cannot be assessed.

## Results

After applying in- and exclusion criteria, 412 cases could be identified. In eight cases (1.9%), a BADO form was available, but was not filled in. In 37 cases (7.0%), the patient files were lost and could not be obtained from the centralized archive. These 37 cases (9%) were excluded from further analyses. Of the 375 cases examined in the current study, 270 (72%) were based of the first BADO version (1998–2004), and 105 (28%) on the revised BADO version (2005–2006). Detailed information on the evaluated variables is provided in **Table 1**.

**Table 1:** Correct classification, sensitivity, and positive predictive value of socio-demographic and clinical variables, and missing information in patient files and the basic documentation (BADO).

	**Correct Classification (%)** **median (min–max)**	**Sensitivity (%)** **median (min–max)**	**PPV (%)** **median (min–max)**	**Missing (%)** **patient files**	**Missing (%)** **BADO**
**Socio-demographic characteristics**					
Date of birth	99.5			0	0
Gender (3)	99.5 (99.0–100)	99.6 (99.2–100)	99.3 (98.5–100)	0	0
Marital status (4)	99.2 (98.4–100)	98.2 (93.3–100)	95.9 (95.3–96.4)	0.8	0.8
Nationality*	96.2 (96.2–100)	91.4 (83.7–99.1)	99.5 (99.5–100)	1.5	1.1
Education (7)	95.3 (80.0–97.6)	72.7 (5.6–87. 2)	81.1 (15.1–100)	4.0	22.9
Occupational situation (7)	97.3 (88.0–99.7)	80.6 (0–90.1)	86.1 (0–100)	3.5	4.3
Living situation (11)	93.1 (65.3–100)	40.0 (0–80.0)	69.0 (0–100)	0	0
Zip code	98.4			1.3	1.1
**Diagnosis according to ICD-10**					
F0: Organic, including symptomatic, mental disorders	98.1			0	0
F1: Mental and behavioral disorders due to psychoactive substance use	86.4			0	0
F2: Schizophrenia, schizotypal and delusional disorders	87.7			0	0
F3: Mood [affective] disorders	91.2			0	0
F4: Neurotic, stress-related and somatoform disorders	95.7			0	0
F5: Behavioral syndromes associated with physiological disturbances and physical factors	98.9			0	0
F6: Disorders of adult personality and behavior	94.1			0	0
F7: Mental retardation	98.9			0	0
F8: Disorders of psychological development	99.5			0	0
F9: Behavioral and emotional disorders with onset occurring in childhood and adolescence	99.7			0	0
**Treatment-related characteristics**	**Correct Classification (%)** **median (min–max)**	**Sensitivity (%)** **median (min–max)**	**PPV(%)** **median (min–max)**	**Missing (%)** **patient files**	**Missing (%)** **BADO**
**Admission and discharge**					
Admission ward	95.2			0	0
Date of admission	97.6			0	0
Discharge ward	92.3			0	0
Date of discharge	89.9			0	0
Treatment duration	88.8			0	0
Sector patient (3)	86.8 (83.8–87.8)	79.4 (78.5–80.3)	83.3 (0–94.8)	1.1	0.3
Type of admission (5)	99.3 (93.1–99.7)	0 (0–73.0)	33.3 (0–83.6)	0	0.8
Type of entry (3)	95.3 (95.2–99.2)	92.0 (87.7–96.2)	83.3 (0–98.1)	0	0.8
Type of discharge (5)	88.4 (63.8–100)	57.1 (12.3–94.1)	76.5 (58.9––81.5)	0	1.3
**Legal aspects and dangerous behavior**					
Legal care (3)	97.3 (95.5–98.1)	78.3 (0–96.6)	78.3 (0–98.3)	0.3	1.6
Forced medication (3)*	95.6 (92.6–97.0)	74.1 (52.9–95.3)	69.2 (0–96.8)	0	3.0
Behavior endangering others**	92.4 (92.4–98.1)	79.8 (66.7–92.9)	40.0 (0–98.9)	0	0
Suicide attempt in past (3)	90.8 (88.7–97.3)	74.6 (58.1–91.2)	46.2 (0–96.3)	0	3.8
Suicidality during admission (3)*	90.0 (43.2–97.8)	74.0 (53.3–94.6)	69.6 (0–94.2)	0	0
Suicidality during treatment (3)*	92.2 (85.6–92.6)	59.9 (31.3–88.6)	33.3 (0–95.7)	0	7.4
Suicide attempt during treatment (3)*	94.4 (91.9–97.4)	48.4 (0–96.9)	0 (0–94.7)	0	2.6

Correct classification (i.e. rate of correctly positive and correctly negative coded items), sensitivity (i.e. probability that an item present in the patient files is correctly coded in the BADO), and positive predictive value (i.e. probability that an item coded in the BADO is indeed present in the patient files) were calculated according to Jaeger et al. (24). Numbers in round brackets indicate the number of available categories for polytomous variables. PPV, positive predictive value. * Variable was only available as part of the paper based routine documentation from 1998–2004 (n = 270) ** variable was only available as part of the paper based routine documentation from 2005–2006 (n = 105).

In the patient files, information on 27 (79.4%) of the examined 34 variables was available. For the remaining variables, information was missing in 0.3% to 4% of the cases. In all cases, routine BADO data were available for 20 (58.8%) of the examined 34 variables. The percentage of missing data for the remaining variables ranged between 0.3% and 22.9%, while only the variables education and suicidality during treatment showing more than 5% missing data.

In general, the overall rate of correct classifications was high, with a median percentage of 86.4% to 99.7% for the examined variables: six variables had a median of 99% and above, 13 variables of <99% to 95%, nine variables of <95% to 90%, and six variables (F1 and F2 diagnoses, date of discharge, treatment duration, sector patient, and type of discharge) had a median below 90%.

Sensitivity, i.e. the fact that a positive rating available in the patient file was also positively recorded in the BADO, was above 75% for eight and <75% but above 50% for six of the examined 17 variables. Living situation, type of admission, and suicide attempt during treatment had the poorest sensitivity. However, there was a large spread of sensitivity over the different categories of the polytomous variables, with a maximum specificity of 90% and above for 12 of the 17 variables and of 80% and above for 16 of the 17 examined variables.

Values for the positive predictive value, i.e. the fact that a positive rating in the BADO was indeed supported by a positive rating in the patient file, were above 75% for nine and <75% but above 50% for three variables. The positive predictive value was worst for type of admission, suicidality during treatment, and suicide attempt during treatment. Again, there was a large range of positive predictive values over categories, with a maximum of 80% and above for all variables.

## Discussion

The current study examined the reliability of paper based routine documentation in psychiatric inpatient care. To our knowledge, it constitutes the first published evaluation of this kind and complements a similar evaluation of the reliability of electronic routine documentation using a comparable instrument (24). Strengths of the study are the large sample size and the cross-check of a random sample of 10% of the patient-file data to guarantee complete and accurate data entry from paper-based patient files.

The finding that 2% of the BADO forms were not filled in and that 7% of the patient files were unavailable underline the importance of at least a rudimentary control for completeness in routine documentation, and the drawbacks of paper-based patient files (3, 4, 18, 19). Paper-based documentation can be easily used, is quick to complete and cost-effective as no IT infrastructure is necessary. However, cumbersome maintenance, searching for relevant information, poor availability of old files, and high storage and conservation costs are well-known problems of paper-based documentation (18, 20). In particular, having no access to 7% of the patient files might lead to adverse consequences, e.g. if a hospital faces legal action. Nevertheless, at least partly paper-based documentation remains reality in psychiatric hospitals, even in health care systems of highly developed countries.

In general, the reliability of routine documentation was found to be adequate in our study. The results concerning correct classification were comparable with findings by Jaeger et al. (24), with values for nationality, occupational situation, living situation, type of entry, and legal care being higher, and values for type of discharge, forced medication, behavior endangering others, and suicidality during admission being lower in our study (24). There were only few variables where sensitivity and positive predictive value were calculated in both studies. Again, the results were comparable for sensitivity, and slightly worse for the positive predictive value in our analysis (24). This could be interpreted as an indication that, at least for the majority of the examined items, methodological improvements like prospective electronic documentation, enforcement of the documentation of mandatory items, and routine checks for completeness and for erroneous data entry don’t seem to have a large impact due to ceiling effects. This pertains to socio-demographic data, disease-related data, and treatment-related data, in particular when data important for administrative, financial, or legal purposes are concerned.

In agreement with Jaeger et al. (24), we found a high spread of values of correct classification, sensitivity, and positive predictive value for different categories of the examined variables (24). This may be the consequence of poor user-friendliness, too complex, and too differentiated categories or category definitions that leave too much uncertainty which category should be chosen (4, 24). Especially rare but clinically highly relevant events like suicide attempts were subject to poor sensitivity and positive predictive value, and the acceptable reliability can largely be attributed to correct negative ratings, i.e. agreement in the routine documentation and paper file that a suicide attempt did not occur (24).

Variables with poor performance that should be considered for revision or elimination from routine documentation include living situation, type of admission, and suicide attempt/suicidality during treatment. In particular, variables that have to be re-checked with clinical documentation (e.g. living situation, education) or taken from registers (e.g. sector patient) have a tendency to be completed without ensuring accuracy of the data and should be viewed critically.

Diagnosis related variables—which were not examined in the study of Jaeger et al. (24)—performed adequately with poorest reliability for F1- and F2-diagnoses. This may have been caused by adjustments of diagnoses at finalization of the documentation when diagnostic processes were finalized and all information was available. Also, differences in the assessments by the treating physician and the supervising psychiatrist could play a role. In addition, there is a well-known diagnostic shift in first-episode psychosis that could contribute to this phenomenon (28). Furthermore—in general—the agreement between multiple psychiatrists diagnosing the same patients is limited, and this may further contribute to this result (29).

## Limitations

Relatively old patient files from 1998 to 2006 were examined and only data from one study site was used, potentially limiting the generalizability of our results. Reliability of the paper-based routine documentation is measured *via* agreement of information recorded in the BADO with information available in paper-based patient files. Whereas these can be considered as mostly complete and representative of the clinical case, documentation quality is inferior compared to, e.g. prospective structured data acquisition for scientific studies. Therefore the current study cannot provide a conclusive answer to the question how reliable routine paper documentation really is, but can be considered to be a valid approximation to this research question. Furthermore, we were only able to examine about 2% of all inpatient cases in the observation period; however, the sample size of the current analysis is larger than in pre-existing literature (30) and can be considered sufficient. Additionally, only first hospitalizations of persons with a psychotic syndrome were included. Although it is unlikely that reliability of routine documentation is influenced by this selection to a relevant degree, this might impair generalizability of our findings, and—in particular—the reliability of diagnosis coding should be re-examined in future studies.

## Conclusion and Recommendations for Further Improvement

In summary, electronic routine documentation has to be considered superior to paper-based routine documentation due to the possibility for automatic checks for completeness, formatting, theoretically possible values, and the possibility to reuse pre-existing administrative data. However, paper-based routine documentation also reaches acceptable reliability in general, but this is dependent on the chosen documentation categories and variables.

Recommendations for further improvement of paper-based routine documentation include implementation of checks for completeness, data plausibility, centralized data entry, controlling and management, definition of the persons responsible for completion, documentation without relevant delay, and clear instructions for completion of individual variables (8, 10). Some of these measures, however, decrease cost-effectiveness of routine documentation and have to be balanced against the possible benefits of improved documentation quality. For all forms of routine documentation, the lack of available time is a limiting factor. Time for completing documentation is missing for other professional activities of the responsible health care staff, i.e. it has to be taken from direct clinical work with the patients, professional training, research, and teaching activities (31).

Based on the present findings, paper-based routine documentation can indeed be used for quality management, organizational development, and health services research, but its limitations have to be kept in mind (4, 22–24).

## Data Availability Statement

The datasets generated for this study are available on request to the corresponding author.

## Ethics Statement

The studies involving human participants were reviewed and approved by Ethik-Kommission der Ärztekammer Hamburg, Hamburg, Germany. Written informed consent for participation was not required for this study in accordance with the national legislation and the institutional requirements.

## Author Contributions

CH designed the study, and CB conducted data entry and management. DF and CH analyzed and interpreted the data, and wrote the initial draft of the paper. CB, KS, DS, EK, SB, and UL revised the paper for important intellectual content. All authors have contributed to, read, and approved the final version of the manuscript. DF had full access to all the data in the study and takes responsibility for the integrity of the data and the accuracy of the data analysis.

## Funding

The authors declare that, expect for income received from their primary employer, no financial support or compensation has been received from any individual or corporate entity over the past 12 months for research of professional service related to this study, and there are no personal financial holdings that could be perceived as constituting a potential conflict of interest.

## Conflict of Interest

The authors declare that the research was conducted in the absence of any commercial or financial relationships that could be construed as a potential conflict of interest.
